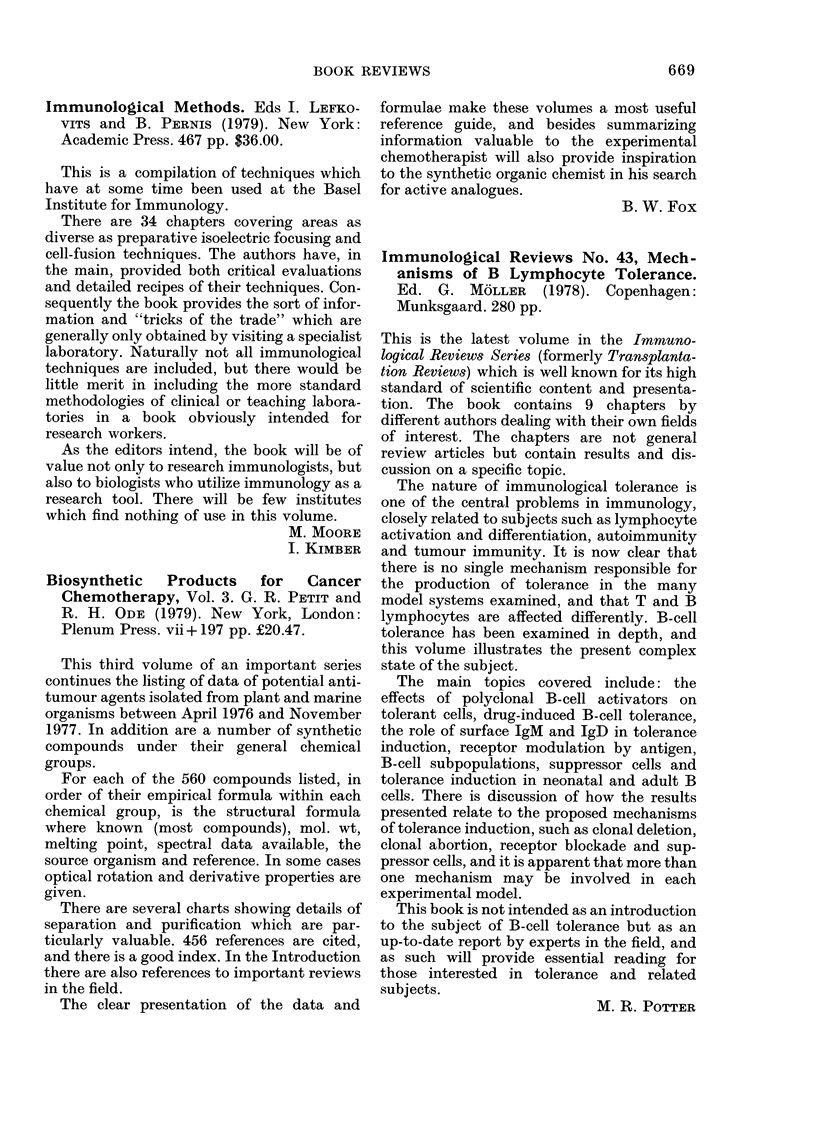# Biosynthetic Products for Cancer Chemotherapy, Vol. 3

**Published:** 1979-10

**Authors:** B. W. Fox


**Biosynthetic Products for Cancer Chemotherapy,** Vol. 3. G. R. PETIT and R. H. ODE (1979). New York, London: Plenum Press. vii+197 pp. £20.47.

This third volume of an important series continues the listing of data of potential antitumour agents isolated from plant and marine organisms between April 1976 and November 1977. In addition are a number of synthetic compounds under their general chemical groups.

For each of the 560 compounds listed, in order of their empirical formula within each chemical group, is the structural formula where known (most compounds), mol. wt, melting point, spectral data available, the source organism and reference. In some cases optical rotation and derivative properties are given.

There are several charts showing details of separation and purification which are particularly valuable. 456 references are cited, and there is a good index. In the Introduction there are also references to important reviews in the field.

The clear presentation of the data and formulae make these volumes a most useful reference guide, and besides summarizing information valuable to the experimental chemotherapist will also provide inspiration to the synthetic organic chemist in his search for active analogues.

B. W. FOX